# Granulomas in Common Variable Immunodeficiency Display Different Histopathological Features Compared to Other Granulomatous Diseases

**DOI:** 10.1007/s10875-024-01817-3

**Published:** 2024-10-07

**Authors:** Astrid C. van Stigt, Jan H. von der Thüsen, Dana A. M. Mustafa, Thierry P. P. van den Bosch, Karishma A. Lila, Disha Vadgama, Martin van Hagen, Virgil A. S. H. Dalm, Willem. A. Dik, Hanna IJspeert

**Affiliations:** 1 Erasmus Medical Center, Laboratory of Medical Immunology, Department of Immunology, Dr. Molewaterplein 40, Rotterdam, 3015 GD The Netherlands; 2 Department of Internal Medicine, Division of Allergy and Clinical Immunology, Erasmus Medical Center, Rotterdam, The Netherlands; 3 Department of Pathology and Clinical Bioinformatics, Erasmus Medical Center, Rotterdam, The Netherlands; 4 The Tumor Immuno-Pathology Laboratory, Department of pathology, Erasmus Medical Center, Rotterdam, The Netherlands; 5 Department of Pulmonary Medicine, Erasmus Medical Center, Rotterdam, The Netherlands

**Keywords:** Common variable immunodeficiency, Granuloma, Histology, Spatial protein expression, Sarcoidosis

## Abstract

Granulomatous disease affects up to 20% of patients with Common Variable Immunodeficiency (CVID). Granulomas are comprised of highly activated immune cells, and emerge in response to antigenic triggers. In CVID granulomas however, the underlying pathophysiology is unclear and the specific trigger remains unknown. Granuloma formation in CVID is often compared to sarcoidosis, although clinical context and prognosis differ, suggesting a different pathogenesis. The aim of this study was to investigate if the cellular organization and proteomics of granulomas in CVID is different from other granulomatous diseases. Therefore, tissue slides from formaldehyde fixed paraffin embedded biopsies obtained from patients with CVID, sarcoidosis, tuberculosis and foreign-material induced pseudo-sarcoidosis were stained with hematoxylin and eosin and assessed for histopathological characteristics. Targeted spatial protein analysis was performed, and immune fluorescent multiplex assays were used to analyze the cellular organization. Histological analysis revealed that CVID granulomas were smaller, less circumscribed, with fewer multinucleated giant cells and minimal fibrosis compared to the other granulomatous diseases. Spatial protein analysis showed that granulomas in all diseases expressed CD68, CD11c, CD44, CD127, and PD-L1. However in CVID, reduced expression of the fibrosis-related protein fibronectin, but enrichment of CD163, CD3 and FAPα inside CVID granulomas was observed. Immunofluorescence analysis conformed a different cellular organization in CVID granulomas with increased influx of neutrophils, macrophages, T and B lymphocytes. In conclusion, granulomas in CVID display a different histological and cellular organization with increased influx of myeloid and lymphoid cells, compared to sarcoidosis, tuberculosis and pseudo-sarcoidosis, indicating a distinct pathogenesis underlying granuloma formation.

**Supplementary Information:**

The online version contains supplementary material available at 10.1007/s10875-024-01817-3.

## Introduction

Common Variable Immunodeficiency (CVID) is an Inborn Error of Immunity characterized by an antibody deficiency. Patients typically suffer from recurrent infections that are relatively well controlled with immunoglobulin replacement therapy and antibiotic treatment [1, 2]. However, a significant number of CVID patients develop additional non-infectious complications, which are associated with increased morbidity and mortality [2, 3]. Granulomatous disease is a serious non-infectious complication that is reported in 8–20% of CVID patients [2–5].

Granulomas are aggregates of highly activated immune cells that encapsulate pathogens or substances that are hard to clear [6–9]. Granulomas can affect every organ, potentially leading to irreversible organ damage if not eradicated. Granulomatous-lymphocytic interstitial lung disease (GLILD), and liver granulomas are associated with reduced survival in CVID patients. This is most likely attributed to the ensuing tissue destruction and complications specific to the affected organs [2–4, 10]. Granulomatous disease in CVID often affects multiple organs per patient, with the lungs, liver and lymph nodes most frequently included [2, 11].

In CVID, the antigenic trigger that induces granulomas is unknown. Moreover, only little information is available on granuloma structure, and cells contributing to granuloma formation. Serum studies in CVID patients with granulomatous diseases revealed elevated levels of proteins suggestive for involvement of T cells, monocyte or macrophages, dendritic cells and neutrophils [12–15]. Also B cell aberrations are observed in CVID patients with granulomatous diseases, such as increased CD21low B cells [2, 12, 13, 16–18]. Interestingly, the anti-CD20 monoclonal antibody rituximab that targets B cells, is reported effective in pulmonary lymphoid hyperplasia and for granuloma remission in CVID patients [15, 19, 20]. CVID granulomas rarely resolve without immune modulatory therapies, while the most effective treatment strategies remain uncertain [5, 11, 21, 22]. The lack of a comprehensive understanding of the pathophysiology underlying granulomas in CVID hinders the development of more effective therapies and specific monitoring tools. This warrants more insight into the immune pathobiology in order to optimize effective clinical care.

Sarcoidosis is a disorder of unknown etiology in which a complex interaction between host, genetics, and environmental triggers results in aberrant immune activation to unidentified antigens [23–26]. Importantly, GLILD can precede the diagnosis of CVID, and since sarcoidosis is more prevalent, initial misdiagnosis of CVID as sarcoidosis is possible [23, 27]. However, in contrast to CVID, spontaneous remission of granulomas occurs in most sarcoidosis patients [5, 23, 28]. Moreover, sarcoid granulomas exhibit histological features characterized by well-circumscribed boundaries with less lymphocyte cuffing and less signs of inflammation [29], while in GLILD lymphocyte infiltration and associated signs of inflammation are frequently reported [10, 29–31]. Interestingly, serum markers indicative for T cell activation (soluble interleukin-2 receptor) as well as macrophage activation (sCD163, sCD206 and Angiotensin-Converting Enzyme (ACE) ) are higher in serum of CVID patients with progressive granulomatous disease than in sarcoidosis patients, supporting the more inflammatory character of CVID granulomas [13–15, 30]. Although these data suggest pathogenic differences between CVID and sarcoid granulomas, some treatment strategies of CVID + GLILD and sarcoidosis are comparable, but unfortunately often less effective for granuloma remission in CVID. This likely relates to insufficient understanding of the immune pathobiology that underlies granuloma formation, maintenance and progression in CVID [11, 21, 24, 30]. Therefore, detailed insight into cellular and molecular processes involved in CVID granulomas, especially in comparison to other granulomatous diseases, is required.

In order to unravel histopathological characteristics specific for CVID granulomas we conducted the current study, in which we compared granulomas from CVID with sarcoid granulomas, tuberculosis (TB) granulomas and granulomas induced by foreign inorganic material (pseudo sarcoidosis: PS). We observed that CVID granulomas are smaller and have lesser pronounced cellular organization, with more inflammation reflected by increased influx of neutrophils, macrophages and lymphocytes as compared to sarcoidosis, TB and PS. We believe that the different histopathological characteristics of CVID granulomas are indicative of a different pathogenesis underlying granuloma formation in CVID.

## Materials and Methods

### Tissue Biopsies

Six formalin fixed paraffin embedded (FFPE) biopsy materials of sarcoidosis, pseudo sarcoidosis (PS) and tuberculosis (TB), were obtained from the department of pathology of the Erasmus MC. For PS, biopsies were included where the known trigger that induced the granuloma formation was inorganic foreign material resulting in sarcoid-like reactions. For Common Variable Immunodeficiency (CVID), only two suitable FFPE biopsies could be obtained from the department of pathology of the Erasmus MC. Further CVID biopsies were obtained via the Dutch public pathology database PALGA. After scanning pathology report conclusions obtained via PALGA, confirming that the patient had CVID, tissue blocks of eligible samples were requested. After initial whole slide scanning of hematoxylin-eosin (HE) stained slides by the involved pathologist (JvdT), 6 biopsies of all disease groups that had (1) sufficient thickness and size required for the tissue slides needed for the spatial protein analysis, immune fluorescent (IF) multiplex stains and H&E stains, and (2) contained > 10 granulomas on the tissue slide for the H&E stain were selected for further analysis. For CVID no lung biopsies were available that fulfilled these criteria therefor we included 4 lymph node- (LN) biopsies and 2 skin- derived biopsies; for sarcoidosis, all biopsies were LN-derived; for PS 5 biopsies were from skin, 1 LN- derived; for TB 3 biopsies were intestinal-, 1 skin-, 1 pleura- and 1 LN-derived. Tissue origin did not contribute to sample clustering (Figure S1).

### Histological, Spatial Proteomics and Immunofluorescent Analysis of Biopsies

We performed histopathologic evaluation and scoring of predetermined histologic hallmarks on hematoxylin-eosin (HE) stained sections by two blinded observers. More detailed information about the scoring can be found in the Extended Methods in the supplementary materials. On subsequent tissue slides, digital spatial protein analysis using GeoMx Digital Spatial Profiler (DSP) (NanoString Technology©, Seattle, WA, USA) was performed [32]. CD68, CD45 and DAPI were used as morphological markers for detecting granulomas in the selected tissue slides. The selected protein modules, targeting 62 proteins, were: Immune cell profiling, Immune activation status, Immune cell typing, PI3K-AKT, and MAPK. On further subsequent tissue slides, immune fluorescent (IF) multiplex assays for CD68, CD11c, CD163, MPO, FAPα, SMA, phospho-ERK1/2, PD-L1, PD-1, CD3, CD4, FOXP3 and CD20 were performed. Scanned slides were analyzed and positive cell detection was quantified using QuPath: Quantitative Pathology and Bioimage analysis software, version 0.4.3 [33]. Extended methods are reported in the supplementary materials.

## Results

### CVID Granulomas Exhibit Smaller size, less well Defined Boundaries and less Distinct Cellular Organization

**Fig. 1:**
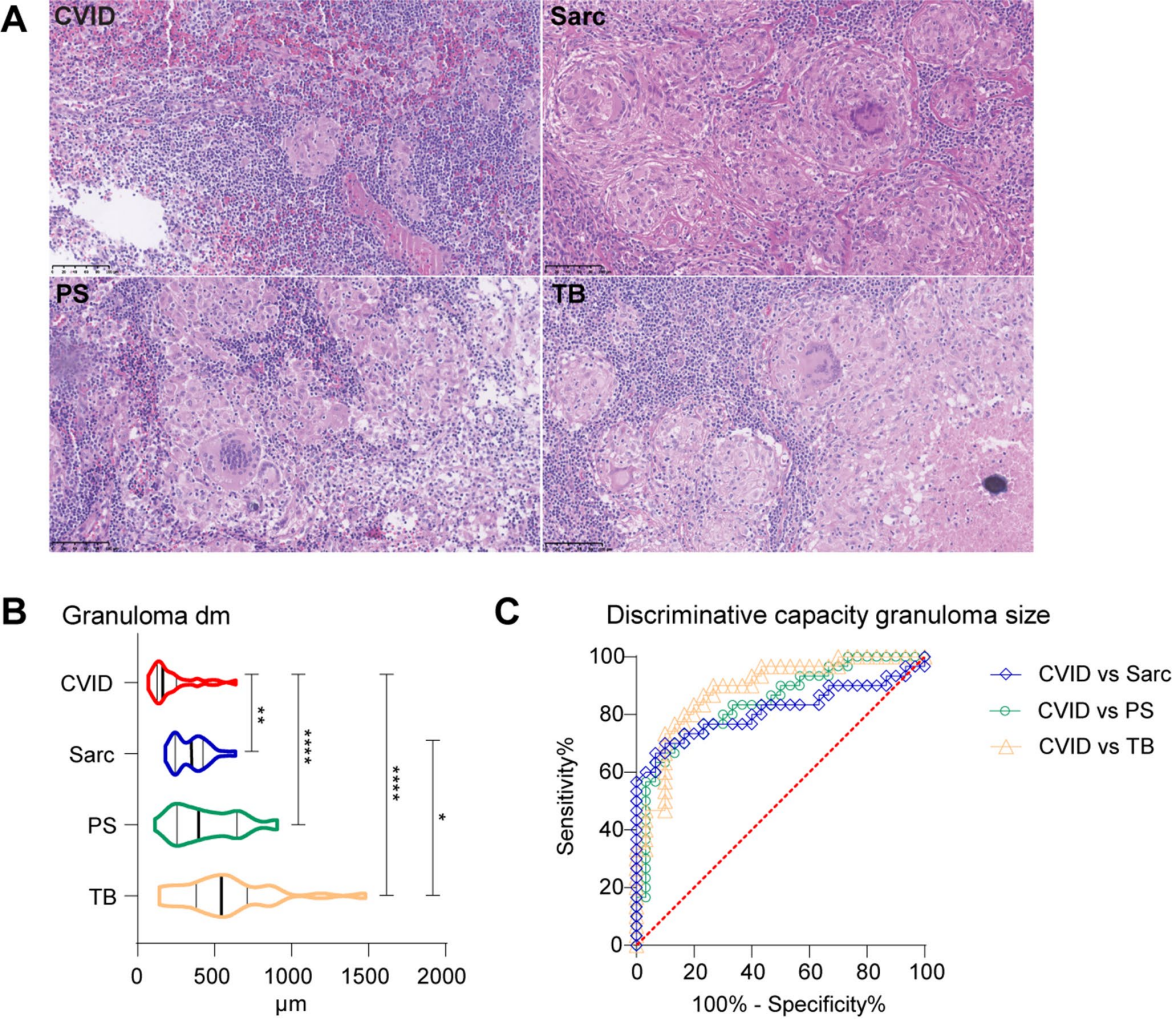
Representative HE stains and granuloma diameter (**A**) 20x Lymph node HE stains of representative biopsies of CVID, sarcoidosis (Sarc), PS and TB. (**B**) Granuloma size per disease group based on the diameter of 30 randomly selected granulomas for each disease group. Median and interquartile ranges are indicated. Kruskal-Wallis with Dunn’s multiple comparison correction, *=*P* < 0.05, **=*P* < 0.01, ****=*P* < 0.0001. (**C**) Discriminative capacity granuloma sizes to discriminate CVID granulomas from sarcoidosis, PS or TB, shown by ROC analysis. See also Table 2

To asses histopathological differences between granulomas in CVID and those from other granulomatous diseases, we performed a blinded scoring of histological parameters on HE stained sections. The biopsies obtained from CVID patients (*n* = 6) were compared to biopsies obtained from sarcoidosis, PS and TB patients (*n* = 6 for all groups). The histological parameters examined included: granuloma size, clustered granulomas, solitary granulomas, circumscribed granulomas, confluent granuloma areas, presence and subtypes of multinucleated giant cells (foreign body giant cells, Touton giant cells, Langhans giant cells, asteroid giant cells), lymphocyte infiltration into granulomas, fibrosis in granulomas and adjacent surroundings, and necrotic areas in granulomas (Table 1; Fig. 1A).

**Table 1:** Histological scoring of granulomas in CVID, sarcoidosis, PS and TB

Histology hallmark	CVID	Sarcoidosis	PS	TB
Original tissue structure affected	2.5 (2–3)	2.5 (2–3)	2 (1–3)	2 (2–3)
Presence of micro granulomas	3***/**/** (2–3)	1 (1–1)	1 (0–2)	0.5 (0–1)
Presence of large granulomas	1****/***/** (0–1)	3 (2–3)	3 (2–3)	3 (2–3)
Clustered granulomas	3 (3–3)	3 (2–3)	2.5 (0–3)	2 (1–3)
Solitary granulomas	1* (0–1)	1.5 (1–3)	2.5 (1–3)	2 (2–3)
Well-circumscribed granulomas	1** (0–2)	3 (2–3)	2 (2–3)	2 (2–3)
Confluent granuloma areas	3* (2–3)	2 (1–3)	1 (0–3)	1.5 (1–3)
Multinucleated giant cells present	0.5 (0–1)	1.5 (1–3)	1 (1–3)	2 (1–3)
Foreign body giant cells	0 (0–0)	0.5 (0–3)	1.5 (0–3)	1 (0–1)
Touton giant cells	0 (0–1)	1 (0–2)	1 (0–2)	1 (0–2)
Langhans giant cells	0.5* (0–3)	3 (1–3)	1.5* (1–3)	3*/* (3–3)
Asteroid body giant cells	0 (0–0)	0 (0–1)	0 (0–3)	0 (0–0)
Lymphocyte infiltration in granuloma	2 (1–2)	1.5 (1–2)	1.5 (1–2)	1 (1–2)
Fibrosis in center of granuloma	0 (0–1)	1** (1–3)	0 (0–1)	1 (1–3)
Fibrosis in adjacent surrounding tissue	0.5** (0–2)	2.5 (1–3)	1 (0–3)	3 (2–3)
Necrotic areas inside granulomas	0 (0–0)	0.5 (0–1)	0 (0–0)	1.5 (0–3)

CVID = Common Variable Immunodeficiency; PS = pseudo sarcoidosis; TB = tuberculosis. Numerical scoring: 0 = not present, 1 = limitedly present, 2 = frequently present, 3 = present throughout slide. A 2way anova, with multiple comparisons test correction using Tukey’s test, comparing the mean of each disease group per histology hallmark was performed; * = *P* ≤ 0.05, ** = *P* ≤ 0.01, *** = *P* ≤ 0.001, **** = *P* ≤ 0.0001

**Table 2:** Discriminative capacity of granuloma size for detecting CVID granulomas

Comparison	Area	cut off size granuloma (µm)	Sensitivity% (95% CI)	Specificity% (95% CI)	LR CVID
CVID vs. Sarc	0.814	< 191	60 (42.3–75.4%)	96.7 (83.3–99.8%)	18
CVID vs. PS	0.842	< 178	56.7 (39.2–72.6%)	96.7 (83.3–99.8%)	17
CVID vs. TB	0.884	< 152	46.7 (30.2–63.9%)	96.7 (83.3–99.8%)	14

CVID = Common Variable Immunodeficiency; PS = pseudo sarcoidosis; TB = tuberculosis. CI = confidence interval. LR CVID = likelihood ratio of being CVID with the cut off size granuloma, sensitivity, specificity and LR derived from ROC analysis

CVID granulomas were significantly smaller than granulomas from the other diseases (Fig. 1B). Moreover, granuloma size served as a discriminating parameter distinguishing CVID from sarcoid granulomas with a granuloma size < 191 μm highly sensitive and specific, as well as from PS or TB granulomas (Fig. 1C; Table 2). CVID granulomas were less solitary and more organized in clusters with confluent areas, were less well circumscribed, and contained less fibrosis (Fig. 1A; Table 1) as opposed to sarcoidosis, PS and TB granulomas, which presented more commonly as solitary and well-circumscribed. Multinucleated giant cells are considered a typical hallmark of granulomas and were indeed commonly observed in granulomas from sarcoidosis, TB and PS [8, 23, 25]. However, granulomas from CVID patients seldom contained multinucleated giant cells (Fig. 1A; Table 1). Overall, granulomatous disease in CVID seems to reflect a less organized cellular process compared to sarcoidosis, PS and TB, and lacks hallmarks linked to granuloma maturation like fibrosis and multinucleated giant cells.

### CVID, Sarcoid, PS and TB Granulomas are Enriched for Proteins Related to Myeloid and T Cell Function

To assess differences in immune biology between CVID granulomas and granulomas from sarcoidosis, PS and TB, we performed digital spatial profiling for proteins associated with immune cell profiles, immune activation¸ PI3K/AKT-signaling and MAPK-signaling both inside the granulomas as well as in their adjacent surrounding (Table S1).

**Fig. 2:**
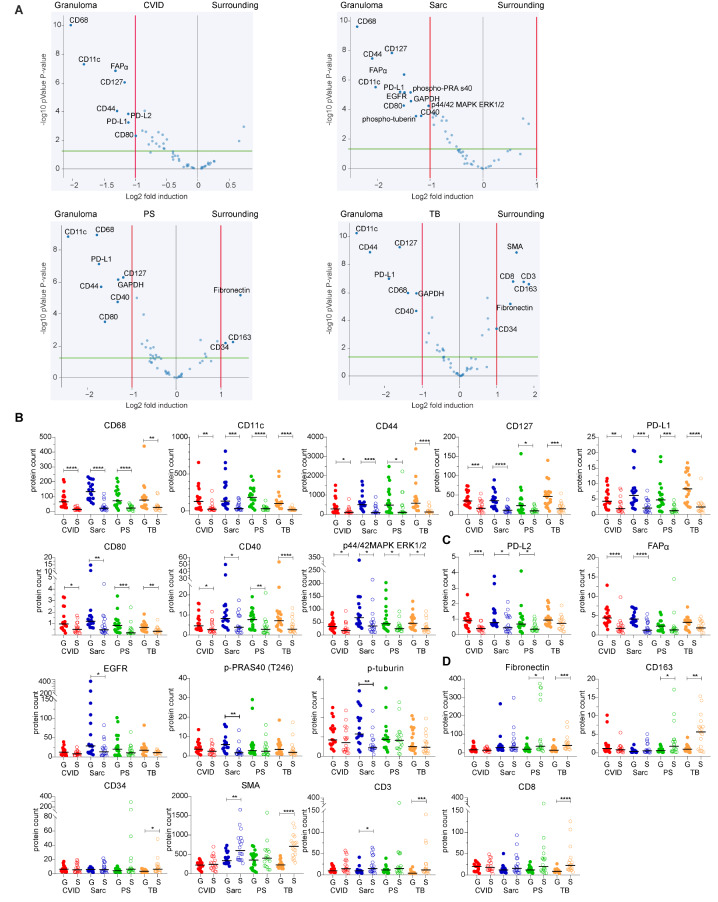
Spatial protein analysis of inter disease comparison granuloma versus surrounding. (**A**) Relative protein expression of 72 granulomas (3 granulomas per each of 6 patients per disease group) and the adjacent surrounding. Fold induction threshold was set to 1 (red line), significance threshold at 1.2 (green line) showing uncorrected P-values. Only significantly or borderline significant targets are indicated. (**B**) Absolute protein counts with median of significant protein expression commonalities in the granulomas of the four diseases. (**C**) Absolute protein counts with median of protein targets enriched in the granulomas versus the surrounding only in a subset of the volcano plots. (**D**) Absolute protein counts with median of protein targets enriched in the surrounding versus the granulomas only in a subset of the volcano plots. Statistical differences only indicated for expression in granuloma versus the surrounding per disease, with statistical testing by Mann-Whitney test with * = *P* ≤ 0.05, ** = *P* ≤ 0.01, *** = *P* ≤ 0.001

No distinct clustering was observed based on diseases group, region, or tissue origin (Figure S1). Comparing the relative protein expression inside the granulomas and their adjacent surroundings per disease, we observed commonalities across all diseases (Fig. 2A). CD68 (expressed by macrophages), CD11c (expressed by dendritic cells), CD127 (expressed by T cells, typically memory T cells), CD44 (expressed by myeloid and epithelial cells; involved in cell adhesion and migration), and PD-L1 (inhibitory molecule, mainly expressed by antigen presenting cells (APCs)) were significantly enriched with increased absolute counts inside the granulomas in all disease groups (Fig. 2A and B). Also the absolute counts of CD80 (expressed by APCs and activated B cells among others; ligand for CD28 and CTLA4, modulating T-cell activation and differentiation), CD40 (costimulatory receptor expressed by APCs, involved in B-cell memory development and germinal center formation), and p44/42MAPK ERK1/2 (MAPK pathway) showed significantly increased counts inside granulomas compared to its surroundings in all disease groups (Fig. 2B), even though not significantly enriched in all disease groups (Fig. 2A).

PD-L2 (which inhibits T-cell proliferation, expressed by APCs among others) was enriched only in CVID granulomas versus their surroundings, and FAPα (fibroblast activating protein-α) appeared enriched in CVID and sarcoid granulomas versus their respective surroundings (Fig. 2A and C). Only sarcoid granulomas had a significant enrichment and protein count of EGFR (epidermal growth factor receptor), and of the PI3K pathway related proteins phospho-PRAs40 and phospho-tuberin (Fig. 2A and C).

For PS and TB, significant enrichment for fibronectin (an extra cellular matrix protein involved in cell adhesion and migration), CD163 (an acute phase regulated receptor expressed by monocytes and macrophages), and CD34 (an adhesion molecule expressed by endothelial cells, fibroblasts and fibrocytes among others) was detected in the surrounding of the granulomas (Fig. 2A and D). SMA (smooth muscle actin, expressed by myofibroblasts mainly), CD3 (general T cell marker), and CD8 (expressed by cytotoxic T cells) were significantly enriched in the surrounding of TB granulomas, with the absolute protein counts of SMA and CD3 also significantly increased in sarcoidosis (Fig. 2A and D). In both CVID and sarcoidosis, no relative enrichment of proteins in the surrounding versus the granulomas was observed (Fig. 2A).

The above data suggest commonalities in the immune pathogenesis of the granulomas. However, we also observed differences, as reflected by the distribution of fibroblast-associated and some hematopoietic markers between the four diseases. This could reflect differences in the local inflammatory milieu between CVID and the other granulomatous diseases.

### CVID Granulomas are Enriched for CD163, CD66b and FAPα and have Reduced Expression of Fibrosis-Associated Proteins in their Surrounding

**Fig. 3:**
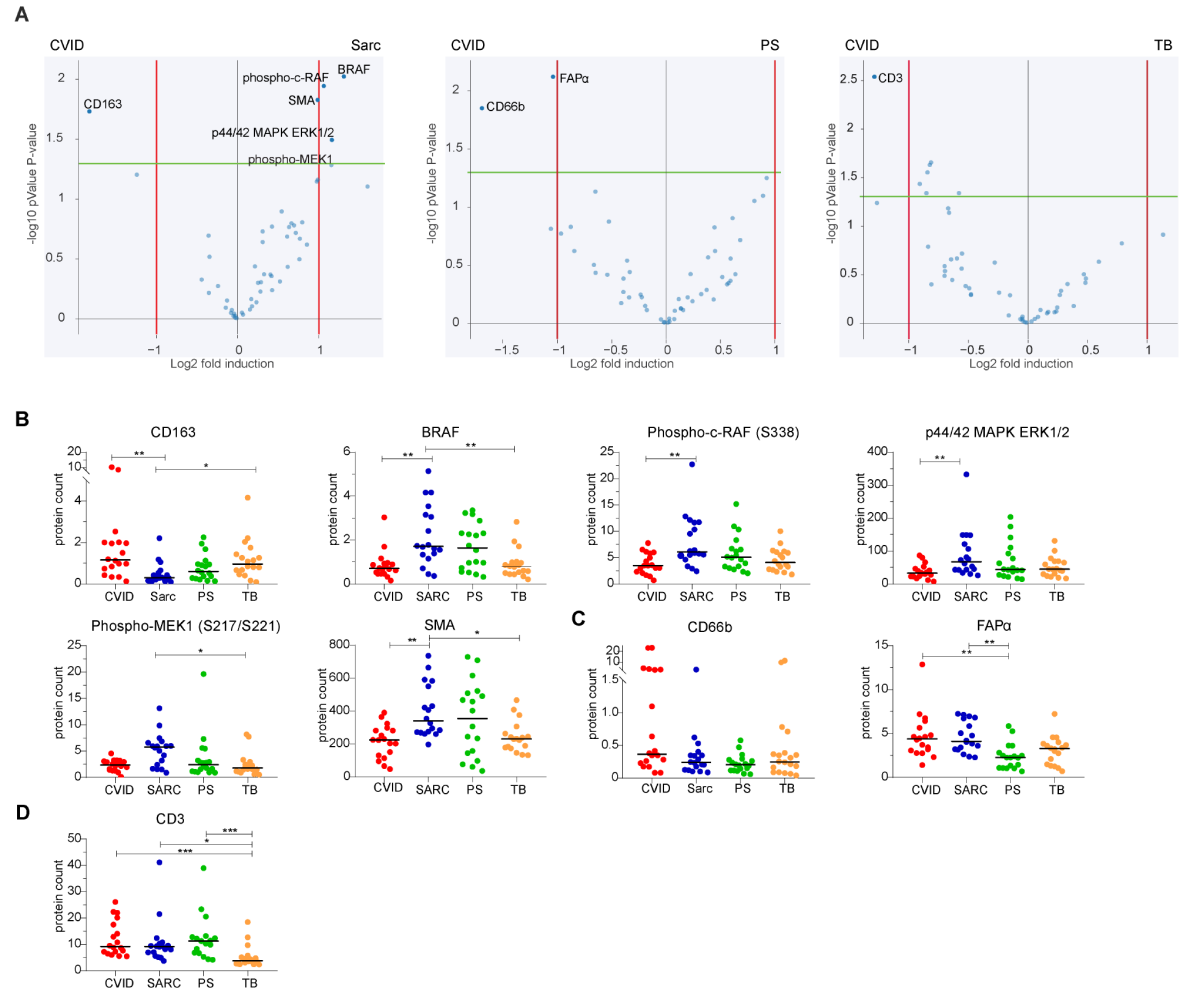
Spatial protein analysis in the granulomas of CVID versus granulomas of sarcoidosis, PS and TB. (**A**) Relative protein expression of 72 granulomas, comparing CVID versus sarcoidosis, or PS or TB, with fold induction threshold set to 1 (red line), significance threshold at 1.2 (green line), showing uncorrected P-values. Only significantly altered or borderline significant targets are indicated. **B-D**) Absolute protein counts with median of significantly enriched protein differences between CVID versus either (**B**) sarcoidosis, (**C**) PS or (**D**) TB. Statistical testing was performed by Kruskall-Walis with Dunn’s multiple comparison correction, * = *P* ≤ 0.05, ** = *P* ≤ 0.01, *** = *P* ≤ 0.001

We next explored differentially expressed proteins inside the CVID granulomas or their surroundings to sarcoidosis, PS and TB. Comparing the protein expression inside the granulomas, CD163 was enriched inside CVID granulomas compared to sarcoid granulomas (Fig. 3A). Absolute protein count of CD163 was highest in CVID, but also in TB the expression was significantly higher compared to sarcoidosis. Interestingly, sarcoid granulomas exhibited a significant enrichment for proteins related to the MAPK signaling pathway when compared to CVID granulomas, including BRAF, phospho-cRAF, p44/42 MAPK ERK1/2, and borderline for phospho-MEK1 (Fig. 3A). These increased MAPK pathway related proteins were unique for sarcoid granulomas (Fig. 3B, Figure S2), even though p44/42 MAPK ERK1/2 was increased in the center of all granulomatous diseases (Fig. 2B). Additionally, SMA was significantly increased in sarcoidosis versus CVID granulomas (Fig. 3A and B).

Compared to PS, CVID granulomas were significantly enriched for CD66b (marker for granulocyte activation, mainly expressed by neutrophils), although not detectable in absolute protein counts. Furthermore, CVID granulomas were significantly enriched for FAPα compared to PS granulomas (Fig. 3A), with absolute counts showing a significant increase in both CVID and sarcoid granulomas compared to PS (Fig. 3C). CD3 was significantly enriched inside CVID granulomas compared to TB (Fig. 3A). The absolute protein counts of CD3 were significantly lower in TB granulomas versus all others (Fig. 3D).

**Fig. 4:**
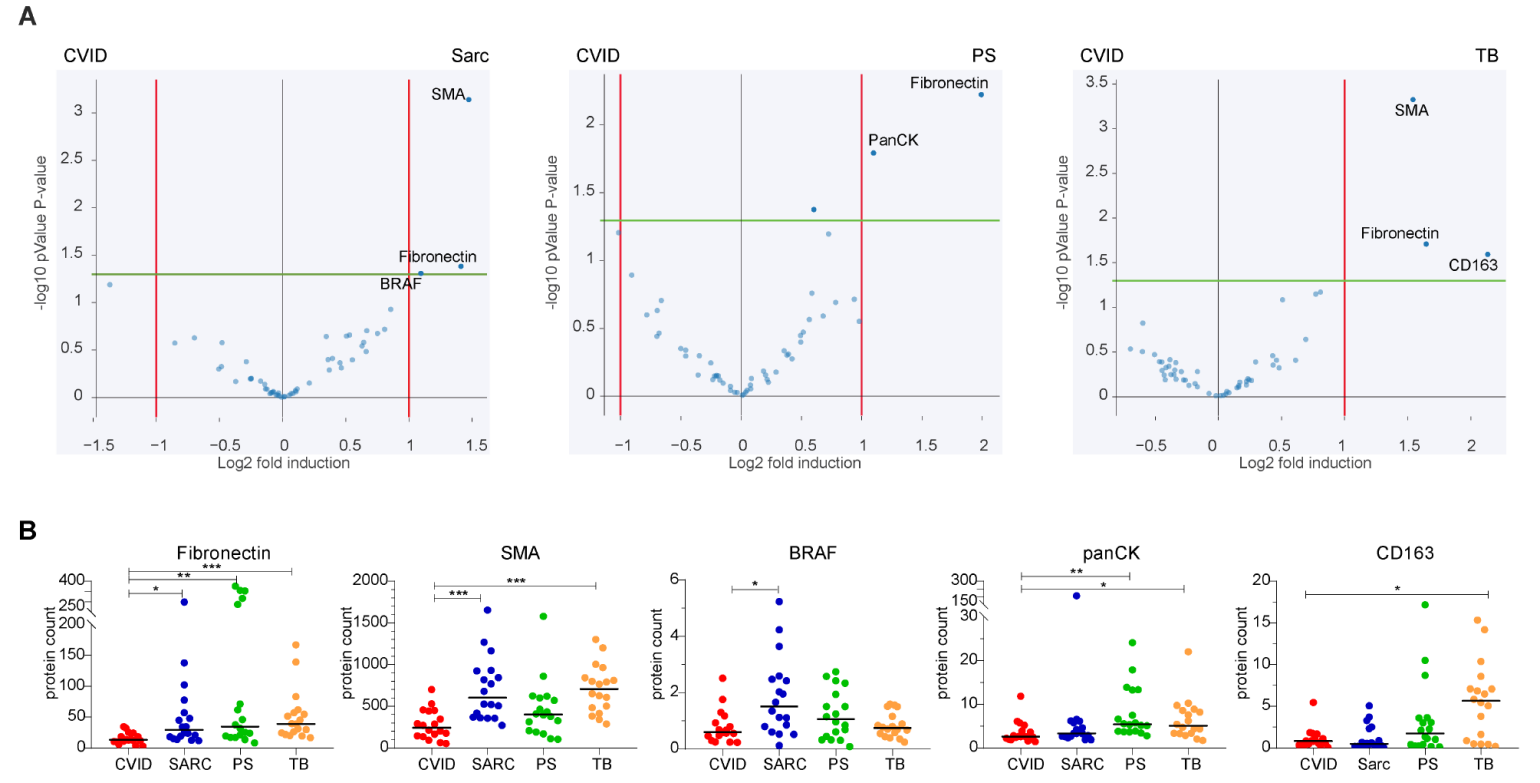
Spatial protein analysis of surrounding areas of granulomas of CVID versus sarcoidosis, PS and TB. (**A**) Relative protein expression of 72 granulomas, comparing CVID versus sarcoidosis, or PS or TB, with fold induction threshold set to 1 (red line), significance threshold at 1.2 (green line), showing uncorrected P-values. Only significantly altered or borderline significant targets are indicated. (**B**) Absolute protein counts with median of significantly depleted protein differences in the surrounding areas of CVID versus sarcoidosis, PS or TB. Statistical testing was performed by Kruskall-Walis with Dunn’s multiple comparison correction, * = *P* ≤ 0.05, ** = *P* ≤ 0.01, *** = *P* ≤ 0.001

Comparing the surroundings revealed some proteins differently expressed in CVID compared to the other granulomatous diseases. Fibronectin was consistently more abundant in the surrounding of the other diseases than CVID, evident from both the significant enrichment and increased protein counts in sarcoidosis, PS, and TB (Figs. 2 and 4A and B). Also, SMA was significantly enriched and increased in the surrounding of sarcoid and TB granulomas as compared to CVID (Fig. 4A and B). BRAF was significantly enriched and increased in the surrounding of sarcoidosis versus CVID (Fig. 4A and B). Cytokeratin (PanCK, expressed by epithelial cells) was enriched in the surrounding of PS granulomas compared to CVID, as for the protein counts which also showed a significant increase in the surrounding of TB compared to CVID (Fig. 4A and B). CD163 was significantly increased only in the surrounding of TB compared to CVID (Fig. 4A and E).

To conclude, targeted protein analysis inside CVID granulomas showed differences compared to the other granulomatous diseases, although these differences were not always unique for CVID. On the other hand, in the surrounding tissue of CVID granulomas lower expression of fibrosis-associated proteins fibronectin and SMA were observed, in line with the limited fibrosis observed for CVID in histological analysis (Fig. 1; Table 1).

### CVID Granulomas Display a Distinct Distribution of Neutrophils, Myeloid Derived Cells and Fibroblasts Compared to Other Granulomatous Diseases

**Fig. 5:**
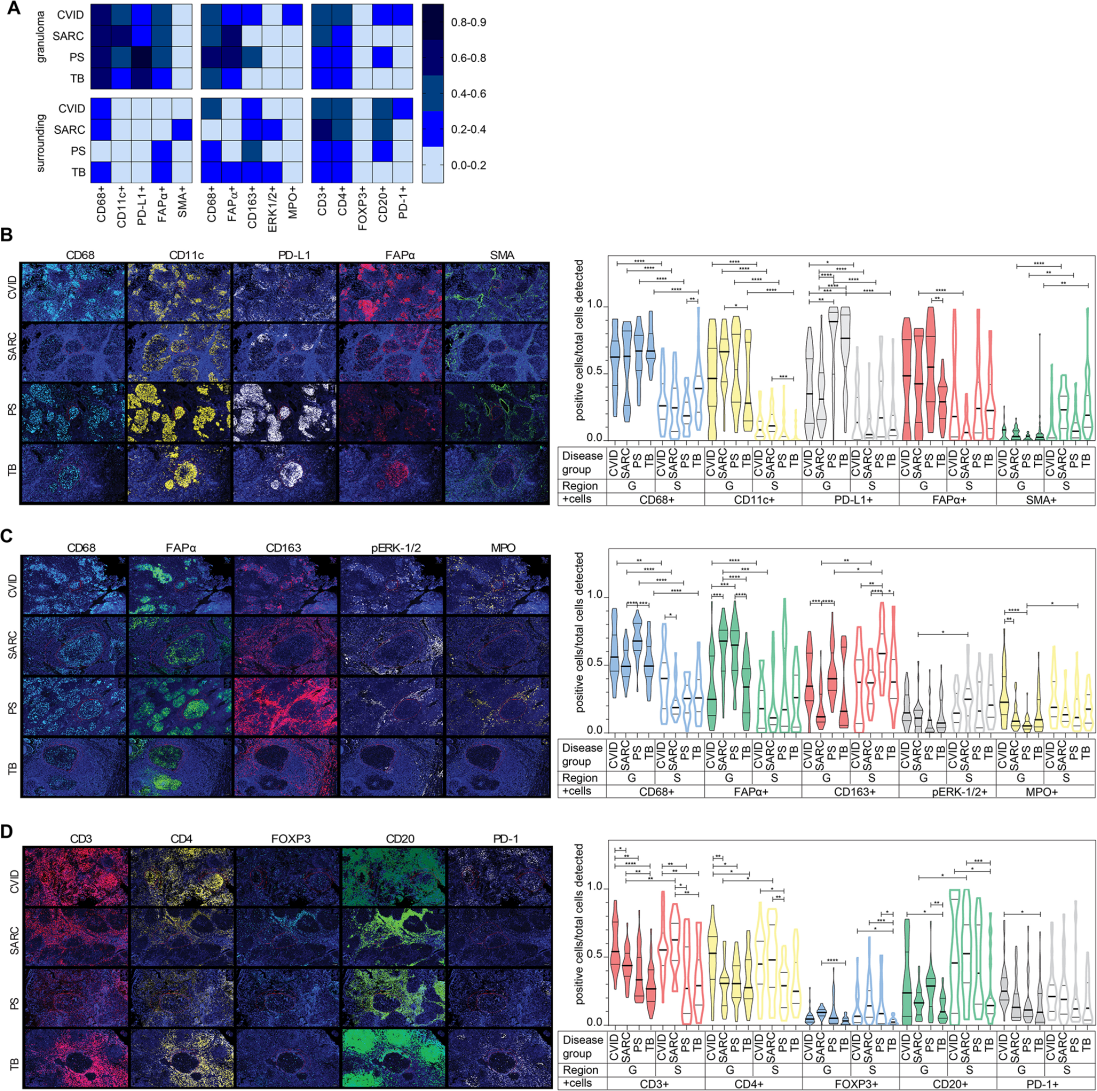
Cellular distribution and quantification of myeloid, fibroblast-like and lymphoid cells in the granulomas and their surroundings. (**A**) Heat map showing the median of positive cell counts of the cellular markers per disease group (CVID, sarcoidosis, PS or TB), normalized towards the total cells detected in that region (granuloma or surrounding). **B-D**) 10x representative immunofluorescence single stains together with DAPI per IF multiplex assay performed, always showing the same biopsy sample per disease of all three IF assays, complemented with the quantified positive cell counts per total cells detected of the indicated regions and markers per disease group, with violin plots showing median and interquartile range; (**B**) DAPI = dark blue, CD68 = aqua, CD11c = yellow, PD-L1 = white, FAPα = red, SMA = green; (**C**) DAPI = dark blue, CD68 = aqua, CD163 = red, pERK1/2 = white, MPO = yellow, FAPa = green; (**D**) DAPI = dark blue, CD3 = red, CD4 = yellow, FOXP3 = aqua, CD20 = green, PD-1 = white. Statistical analysis with 2way anova, with Tukey’s multiple comparisons test, * = *P* ≤ 0.05, ** = *P* ≤ 0.01, *** = *P* ≤ 0.001

To better understand the cellular organization and distribution of CVID granulomas and their surrounding tissue, we conducted three immunofluorescence (IF) multiplex assays on subsequent biopsy slides, to observe how myeloid cells (CD68+, CD163+, CD11c+, and MPO + cells) and fibroblasts-like cells (FAPα + cells) were distributed (Fig. 5). We also included certain protein targets from the previous spatial protein analysis (PD-L1+, SMA+, p-ERK1/2+) (Fig. 5, Figure S3).

Consistent with the digital spatial protein analysis, CD11c + and PD-L1 + cells were mainly localized inside the granulomas (Fig. 5A and B), and were significantly more abundant there than in the surrounding tissue for all diseases (Fig. 5B). FAPα + cells were mainly observed in the granulomas of all diseases groups (Fig. 5A-C), which corresponded with the trend of increased FAPα expression we observed in the spatial analysis (Fig. 2C). This difference however, was only significant for sarcoidosis in the two multiplex assays containing FAPα (Fig. 5B and C). Interestingly, we observed two different staining patterns for FAPα, that were neither disease nor organ specific; in some biopsies FAPα + cells were observed inside the granulomas, while in other biopsies FAPα + cells formed a ring surrounding the granulomas (Fig. 5B and C, Figure S3).

As observed before (Fig. 2D), SMA was increased in the surrounding tissue of sarcoid and TB granulomas compared to CVID and PS, with the majority of observed SMA + cells located around vessel structures (Fig. 5B).

In all disease groups, the number of CD163 + cells was higher in the surrounding than inside the granulomas, with the CD163 + cells inside the CVID and PS granulomas even more abundant than in the sarcoid or TB granulomas (Fig. 5C). Although MAPK-associated proteins were enriched in sarcoidosis versus CVID (Figs. 2 and 3, Figure S2), no significant differences of phospho-ERK1/2 were detected between CVID and sarcoidosis (Fig. 5C). Only a significant increase of phospho-ERK1/2 + cells in sarcoid granulomas versus their surroundings was detected (Fig. 5C).

Since we observed a significant enrichment of CD66b in granulomas of CVID compared to PS, we wanted to investigate this further (Fig. 3). Therefore, we included myeloperoxidase (MPO) as a neutrophil marker. In all disease but CVID, MPO + cell counts were higher in the surroundings versus the granulomas. In CVID granulomas, MPO + cells were clearly increased compared to the granulomas of the other diseases, being significant compared to sarcoidosis and PS (Fig. 5A and C). Similar to the distribution of CD163 + cell counts, the MPO + cell counts in the granulomas and their surroundings were comparable for CVID (Fig. 5B and C).

The IF multiplex assays corresponded with the observations from the spatial protein analysis for CD11c, PDL1, CD163, CD66b and SMA. Compared to the other granulomatous diseases, CVID granulomas contained more CD163 + and MPO + cells within the granulomas as well as in the adjacent surrounding, suggesting that neutrophils and M2-like macrophages might contribute to the pathogenesis of CVID granulomas.

### CVID Granulomas and Their Surrounding Areas show a Different T- and B-Cell Distribution

Considering the importance of lymphoid cells in granuloma formation, we included CD3, CD4, FOXP3 (expressed by T-regulatory cells), CD20 (expressed by B cells) and PD-1 (Programmed Death receptor 1; innate and adaptive inhibitor, expressed on activated T- and B cells, DCs and macrophages) into the IF multiplex analysis (Fig. 5A and D). The IF stains clearly showed presence of CD3 + cells (which were mainly CD4+) in CVID granulomas, as opposed to the granulomas of sarcoidosis, PS and TB (Fig. 5D and Figure S3). In TB, CD3 + and CD4 + cells formed a ring-like structure surrounding the granulomas (Fig. 5D). Overall, in CVID the CD3 + and CD4 + cell counts were comparable between the granulomas and their surroundings (Fig. 5D). The FOXP3 + cell counts were overall low. Especially in the surrounding of TB granulomas, significantly fewer FOXP3 + cells were identified compared to all other diseases (Fig. 5D). Similar to CD3 + cells, CD20 + cells seemed to localize at the border of the granulomas in sarcoidosis, PS and TB granulomas. In CVID granulomas CD20 + cells were abundant, especially compared to sarcoid and TB granulomas (Fig. 5D). Although not significant (except for CVID granulomas versus TB granulomas), the presence of PD-1 + cells seemed more pronounced inside CVID granulomas and their surroundings compared to the other granulomatous diseases (Fig. 5A and D).

Also concerning the lymphoid cells, CVID granulomas seemed less organized. Also the presence of CD3+, CD4 + and CD20 + cells in CVID granulomas could be indicative of a lymphoid inflammatory milieu different from that in sarcoidosis, PS and TB granulomas.

## Discussion

**Fig. 6:**
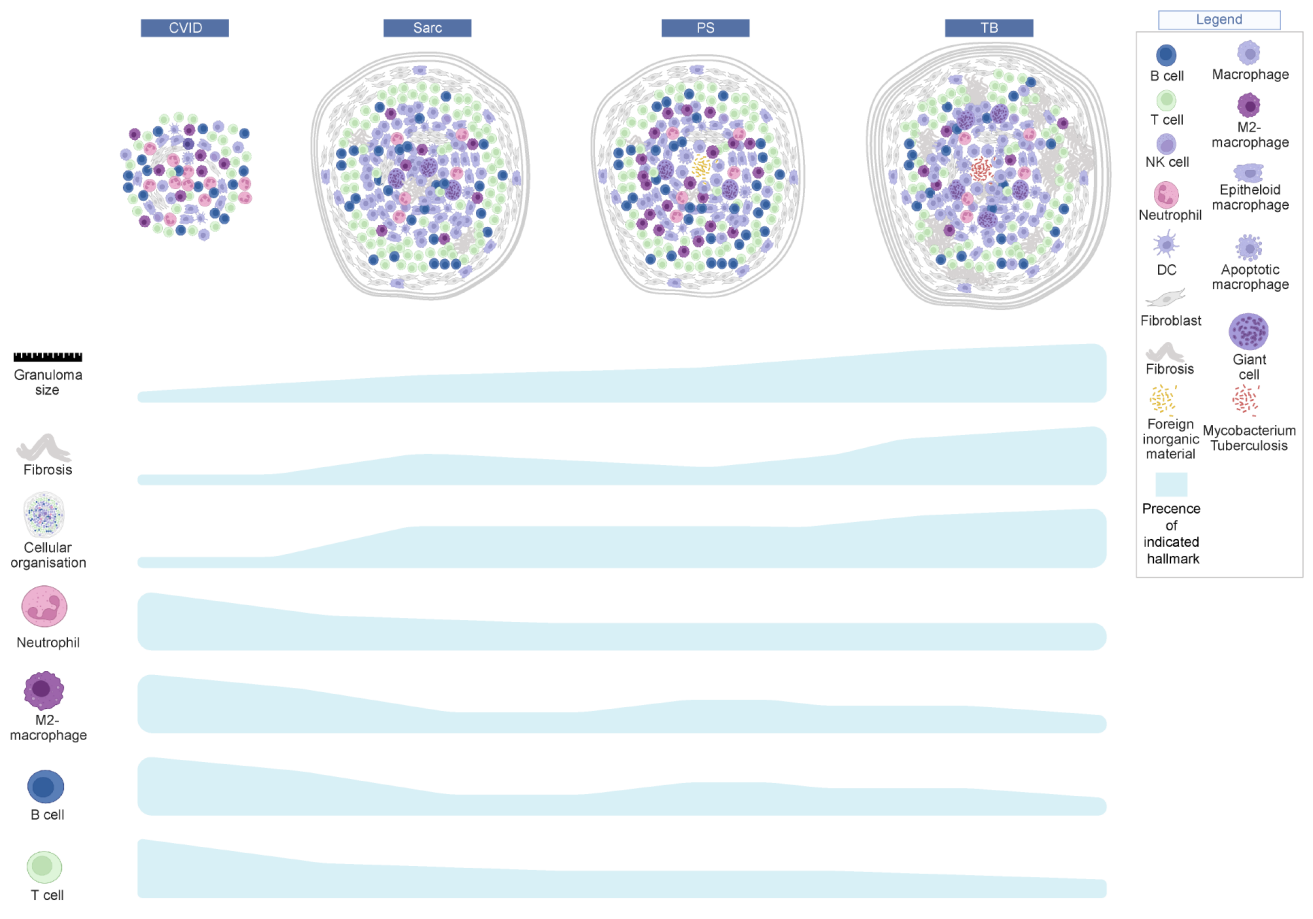
Summary of immune histologic hallmarks of CVID, sarcoidosis, pseudo sarcoidosis (PS) and tuberculosis (TB) granulomas. The graphical overview shows the investigated hallmarks that differed distinctively in CVID. The observed presence of these hallmarks in CVID, sarcoidosis, PS or TB granulomas are indicated with the blue gradient. Created with www.Biorender.com

Understanding the immune histopathogenesis of granulomatous CVID is crucial for improving clinical care and treatment outcomes. We observed CVID granulomas to be smaller, less well defined and to contain hardly any fibrosis or multi-nucleated giant cells (Fig. 1). This different and less well defined cellular organization is also reflected by the distribution in CVID granulomas and their surroundings of CD68+, CD163+, MPO+, CD3+, CD4 + and CD20 + cells (Figs. 2, 3, 4 and 5). Furthermore, the increased myeloid and lymphoid cell influx indicates that CVID granulomas are more inflammatory (Fig. 5). Overall, we believe CVID granulomas differ in their histological hallmarks and cellular distribution, when compared to sarcoidosis, pseudo sarcoidosis and tuberculosis granulomas. Although these four granulomatous diseases each present their own typical immune and histological profile, CVID granulomas have the most diverging profile when compared to the other three diseases (Fig. 6).

Histology analysis is a pivotal step in the clinical work-up of patients suspected of granulomatous disease. Our histology analysis showed that CVID granulomas were clearly distinguishable from the other granulomatous diseases, based on relatively simple histological hallmarks (less circumscribed and more confluent, almost without multinucleated giant cells and fibrosis) and overall smaller granuloma size (Fig. 1; Table 1). Especially the observed differences in histological and lymphoid distribution between CVID and sarcoid granulomas were evident, as also reported by others [29, 34]. Particularly in averting misdiagnosis of sarcoidosis in granulomatous CVID, a granuloma size < 191 μm proved a highly sensitive and specific diagnostic tool, that could be easily implementable in clinical practice (Table 2). These results are in line with the large (16 patients) histology study performed by Rao et al. in CVID patients with GLILD performed so far [29]. They observed that granulomas ranged from well to poorly organized, were non-necrotizing, with varying degrees of inflammation, with CD4 + T- and B cells being the most abundant cells, and with a lack of regulatory T cells [29]. Rao et al. did however observe interstitial fibrosis in 12 out of 16 CVID lung biopsies and reported epithelioid histiocytic and multinucleated giant cells. Fibrosis and multinucleated giant cells were not prominently observed in our CVID biopsies. However, our CVID biopsies are LN and skin derived and thus lack the context of ILD, which might explain these reported differences. Alterations in peripheral blood and serum markers related to T cells are reported [5, 12–15, 31, 35–37]. Of CVID patients with GLILD or progressive granulomatous disease, lower counts of CD4 + and CD8 + T cells are reported also when compared to those without granulomatous disease or sarcoidosis [13, 14, 31, 35, 36]. These CVID patients with granulomatous disease also exhibit a reduced CD4+/CD8 + ratio, similar to what is observed in BALF [5, 14, 37]. Notably, some report a greater reduction of CD8 + T cells alongside a moderate reduction in CD4 + T cells in granulomatous CVID [14, 35]. This could suggest that T cells migrate towards the granuloma-containing tissues in CVID, maybe more so than in sarcoidosis, in line with our histology observations. In serum of CVID + GLILD patients, elevated serum levels of CD6, CD28, soluble sIL-2r, sTIM-3, IFN-γ, and TNF are measured, indicating Th1 cell activation and exhaustion, suggestive for the chronic inflammation and immune dysregulation, and possibly prolonged antigen exposure, of which granulomas could be a resulting complication [12, 13].Krausgruber et al. recently demonstrated that sarcoid granulomas share many similarities with tertiary lymphoid structures, albeit being insufficiently self-limiting [26]. Tertiary lymphoid structures are also reported in CVID patients with pulmonary lymphoid hyperplasia, wherein poorly formed granulomas could be found [19]. We observed a different and less well defined cellular organization of the CVID granulomas, on histological and cellular level, when compared to the sarcoidosis biopsies included in our study. This might indicate that granulomas in CVID are some form of aberrantly organized lymphoid structures, with potentially less regulation of self-limiting pathways as described for sarcoidosis.

The higher counts of CD163 + cells in CVID granulomas versus sarcoid granulomas aligns with our previous observation of higher serum levels of CD163, along with CD206, in patients with granulomatous CVID compared to sarcoidosis, and thus corroborates a prominent role for macrophage activation in CVID granulomas [14]. That macrophages are highly activated in granulomatous CVID is also shown by Fraz et al., whom detected increased sCD163 in serum of CVID + GLILD patients versus CVID with other non-infectious complications and infections only [13]. Generally CD163 is linked to the anti-inflammatory M2 subtype, although this macrophage sub-classification is not stringent, and could support the more chronic inflammatory milieu of CVID granulomas [38]. Of note, other protein markers associated with M2 phenotypes were not present in our spatial protein analysis panels. The observed targeted therapy potential of CD163 + macrophages in animal studies, where glucocorticosteroid where directly delivered to CD163 + macrophages, together with its exclusive monocyte and macrophage expression, makes CD163 an interesting marker for targeted therapy for granulomatous CVID [38–42]. The significant increase of MPO + cells in CVID granulomas is intriguing, since recurrent infections are a hallmark of all CVID patients. Thus, it should be considered that abundant MPO + cells in the CVID granulomas could be induced by a bacterial antigenic trigger to which CVID patients are frequently exposed. In blood, Maglione et al. detected a very slight increase in neutrophil counts of CVID patients with progressive GLILD, compared to CVID with stable or no GLILD, with no noteworthy differences in monocyte counts or WBC [43]. However, slightly decreased MPO levels in serum of CVID + GLILD patients versus CVID with other complications or only infections are also reported making the contribution of neutrophils to the pathogenesis of granulomas in CVID a potential subject for further investigation [13]. 

A role for B cells in non-infectious complications in CVID, including granulomatous disease, has frequently been suggested [2, 3, 13, 16, 18]. Rituximab, a B-cell depleting monoclonal antibody, can have a beneficial effect on CVID patients with pulmonary lymphoid hyperplasia [19, 20]. Considering that expansion of CD21low B cells is associated with CVID + GLILD, and our observed increased influx of CD20 + B cells in CVID granulomas, suggest that B cells might be important for sustaining CVID granulomas [5, 44].

Our data indicate that, although certain immune mechanisms and cellular components are shared, granulomas in CVID are different from the granulomas in sarcoidosis, PS and TB. Overall, CVID granulomas are less well organized, with CD163 + macrophages, neutrophils and B cells potentially important for the maintenance of the granulomas and potential targets for mechanism-based therapies. Additionally, our tissue analysis further shows that routine histology analysis alone can be helpful in discriminating between CVID and sarcoidosis.

While our study contributes to the understanding of granulomas in CVID, especially in comparison to other granulomatous diseases, certain limitations must be addressed. For the CVID biopsies collected via the PALGA database, it was not possible to obtain additional clinical information regarding duration of the disease, other relevant complications, or prescribed immune modulatory therapies since only anonymized biopsy samples were provided. Another limitation was the variation in anatomical origin of the included biopsies. For CVID, inclusion of lung-derived biopsies would have been informative, as GLILD is a distinctive granulomatous disease. Unfortunately, we were not able to include suitable lung biopsies for the CVID group. For sarcoidosis, all biopsies were LN-derived. Fortunately, we observed no clustering in the spatial protein analysis of biopsies obtained from the same anatomical origins, suggesting that the anatomical origin of the biopsies did not introduce a bias in our study. Another technical limitation is the preselected set of protein targets in the Nanostring panels, which limited the further characterization of specific cell types. Also, we are aware that we validated our spatial protein analysis by IF multiplex assays on the same samples. Unfortunately it was not possible to obtain a new independent validation cohort.

## Electronic Supplementary Material

Below is the link to the electronic supplementary material.

Supplementary Material 1

Supplementary Material 2

Supplementary Material 3

Supplementary Material 4

## Data Availability

No datasets were generated or analysed during the current study.

## References

[1] Seidel MG, Kindle G, Gathmann B, Quinti I, Buckland M, van Montfrans J, et al. The European Society for Immunodeficiencies (ESID) Registry Working definitions for the clinical diagnosis of inborn errors of immunity. J Allergy Clin Immunol Pract. 2019;7(6):1763–70. 30776527 10.1016/j.jaip.2019.02.004

[2] Ho HE, Cunningham-Rundles C. Non-infectious complications of common variable immunodeficiency: updated clinical spectrum, Sequelae, and insights to Pathogenesis. Front Immunol. 2020;11:149. 32117289 10.3389/fimmu.2020.00149 PMC7025475

[3] Resnick ES, Moshier EL, Godbold JH, Cunningham-Rundles C. Morbidity and mortality in common variable immune deficiency over 4 decades. Blood. 2012;119(7):1650–7. 22180439 10.1182/blood-2011-09-377945 PMC3286343

[4] Bates CA, Ellison MC, Lynch DA, Cool CD, Brown KK, Routes JM. Granulomatous-lymphocytic lung disease shortens survival in common variable immunodeficiency. J Allergy Clin Immunol. 2004;114(2):415–21. 15316526 10.1016/j.jaci.2004.05.057

[5] Verbsky JW, Routes JM. Sarcoidosis and common variable immunodeficiency: similarities and differences. Semin Respir Crit Care Med. 2014;35(3):330–5. 25007085 10.1055/s-0034-1376862

[6] Cronan MR. In the Thick of it: formation of the Tuberculous Granuloma and its effects on host and therapeutic responses. Front Immunol. 2022;13:820134. 35320930 10.3389/fimmu.2022.820134 PMC8934850

[7] Cronan MR, Beerman RW, Rosenberg AF, Saelens JW, Johnson MG, Oehlers SH, et al. Macrophage epithelial reprogramming underlies mycobacterial Granuloma formation and promotes infection. Immunity. 2016;45(4):861–76. 27760340 10.1016/j.immuni.2016.09.014 PMC5268069

[8] Pagan AJ, Ramakrishnan L. The formation and function of Granulomas. Annu Rev Immunol. 2018;36:639–65. 29400999 10.1146/annurev-immunol-032712-100022

[9] Todorov TI, de Bakker E, Smith D, Langenberg LC, Murakata LA, Kramer MHH et al. A case of silicone and sarcoid granulomas in a patient with highly cohesive silicone breast implants: a histopathologic and laser Raman Microprobe Analysis. Int J Environ Res Public Health. 2021;18(9). 10.3390/ijerph18094526 PMC8123188 33923240

[10] Patel S, Anzilotti C, Lucas M, Moore N, Chapel H. Interstitial lung disease in patients with common variable immunodeficiency disorders: several different pathologies? Clin Exp Immunol. 2019;198(2):212–23. 31216049 10.1111/cei.13343 PMC6797878

[11] van Stigt AC, Dik WA, Kamphuis LSJ, Smits BM, van Montfrans JM, van Hagen PM, et al. What works when treating Granulomatous Disease in genetically undefined CVID? A systematic review. Front Immunol. 2020;11:606389. 33391274 10.3389/fimmu.2020.606389 PMC7773704

[12] Berbers RM, Drylewicz J, Ellerbroek PM, van Montfrans JM, Dalm V, van Hagen PM, et al. Targeted proteomics reveals inflammatory pathways that classify Immune Dysregulation in Common Variable Immunodeficiency. J Clin Immunol. 2021;41(2):362–73. 33190167 10.1007/s10875-020-00908-1 PMC7858548

[13] Fraz MSA, Michelsen AE, Moe N, Aalokken TM, Macpherson ME, Nordoy I, et al. Raised serum markers of T cell activation and exhaustion in granulomatous-lymphocytic interstitial lung disease in common variable immunodeficiency. J Clin Immunol. 2022;42(7):1553–63. 35789314 10.1007/s10875-022-01318-1 PMC9255534

[14] van Stigt AC, Dalm V, Nagtzaam NMA, van Hagen PM, Dik WA. Soluble Interleukin-2 Receptor/White blood cell ratio reflects Granulomatous Disease Progression in Common Variable Immune Deficiency. J Clin Immunol. 2023;43(8):1754–7. 37542638 10.1007/s10875-023-01560-1 PMC10661782

[15] van Stigt AC, Dalm V, Nagtzaam NMA, van Rijswijk DA, Barendregt BH, van Hagen PM, et al. Soluble Interleukin-2 receptor is a promising serum biomarker for Granulomatous Disease in Common Variable Immune Deficiency. J Clin Immunol. 2021;41(3):694–7. 33404971 10.1007/s10875-020-00947-8 PMC7921039

[16] Isnardi I, Ng YS, Menard L, Meyers G, Saadoun D, Srdanovic I, et al. Complement receptor 2/CD21- human naive B cells contain mostly autoreactive unresponsive clones. Blood. 2010;115(24):5026–36. 20231422 10.1182/blood-2009-09-243071 PMC3373152

[17] Keller B, Warnatz K. T-bet(high)CD21(low) B cells: the need to unify our understanding of a distinct B cell population in health and disease. Curr Opin Immunol. 2023;82:102300. 36931129 10.1016/j.coi.2023.102300

[18] Sanchez-Ramon S, Radigan L, Yu JE, Bard S, Cunningham-Rundles C. Memory B cells in common variable immunodeficiency: clinical associations and sex differences. Clin Immunol. 2008;128(3):314–21. 18620909 10.1016/j.clim.2008.02.013 PMC2692232

[19] Maglione PJ, Ko HM, Beasley MB, Strauchen JA, Cunningham-Rundles C. Tertiary lymphoid neogenesis is a component of pulmonary lymphoid hyperplasia in patients with common variable immunodeficiency. J Allergy Clin Immunol. 2014;133(2):535–42. 24131823 10.1016/j.jaci.2013.08.022 PMC4109033

[20] Tessarin G, Baronio M, Gazzurelli L, Rossi S, Chiarini M, Moratto D, et al. Rituximab Monotherapy is effective as first-line treatment for granulomatous lymphocytic interstitial lung disease (GLILD) in CVID patients. J Clin Immunol. 2023;43(8):2091–103. 37755605 10.1007/s10875-023-01587-4 PMC10661825

[21] Smits B, Goldacker S, Seneviratne S, Malphettes M, Longhurst H, Mohamed OE, et al. The efficacy and safety of systemic corticosteroids as first line treatment for granulomatous lymphocytic interstitial lung disease. J Allergy Clin Immunol. 2023;152(2):528–37. 36587851 10.1016/j.jaci.2022.12.813

[22] Lamers OAC, Smits BM, Leavis HL, de Bree GJ, Cunningham-Rundles C, Dalm V, et al. Treatment strategies for GLILD in common variable immunodeficiency: a systematic review. Front Immunol. 2021;12:606099. 33936030 10.3389/fimmu.2021.606099 PMC8086379

[23] Grunewald J, Grutters JC, Arkema EV, Saketkoo LA, Moller DR, Muller-Quernheim J, Sarcoidosis. Nat Rev Dis Primers. 2019;5(1):45. 31273209 10.1038/s41572-019-0096-x

[24] Grutters JC, van den Bosch JM. Corticosteroid treatment in sarcoidosis. Eur Respir J. 2006;28(3):627–36. 16946094 10.1183/09031936.06.00105805

[25] Kraaijvanger R, Janssen Bonas M, Vorselaars ADM, Veltkamp M. Biomarkers in the diagnosis and prognosis of sarcoidosis: current use and future prospects. Front Immunol. 2020;11:1443. 32760396 10.3389/fimmu.2020.01443 PMC7372102

[26] Krausgruber T, Redl A, Barreca D, Doberer K, Romanovskaia D, Dobnikar L, et al. Single-cell and spatial transcriptomics reveal aberrant lymphoid developmental programs driving granuloma formation. Immunity. 2023;56(2):289–306. e7. 36750099 10.1016/j.immuni.2023.01.014 PMC9942876

[27] Arkema EV, Cozier YC. Sarcoidosis epidemiology: recent estimates of incidence, prevalence and risk factors. Curr Opin Pulm Med. 2020;26(5):527–34. 32701677 10.1097/MCP.0000000000000715 PMC7755458

[28] Nunes H, Uzunhan Y, Gille T, Lamberto C, Valeyre D, Brillet PY. Imaging of sarcoidosis of the airways and lung parenchyma and correlation with lung function. Eur Respir J. 2012;40(3):750–65. 22790910 10.1183/09031936.00025212

[29] Rao N, Mackinnon AC, Routes JM. Granulomatous and lymphocytic interstitial lung disease: a spectrum of pulmonary histopathologic lesions in common variable immunodeficiency–histologic and immunohistochemical analyses of 16 cases. Hum Pathol. 2015;46(9):1306–14. 26138782 10.1016/j.humpath.2015.05.011 PMC4554947

[30] Bouvry D, Mouthon L, Brillet PY, Kambouchner M, Ducroix JP, Cottin V, et al. Granulomatosis-associated common variable immunodeficiency disorder: a case-control study versus sarcoidosis. Eur Respir J. 2013;41(1):115–22. 22903958 10.1183/09031936.00189011

[31] Buso H, Discardi C, Bez P, Muscianisi F, Ceccato J, Milito C et al. Sarcoidosis versus Granulomatous and lymphocytic interstitial lung disease in common variable immunodeficiency: a comparative review. Biomedicines. 2024;12(7). 10.3390/biomedicines12071503 PMC11275071 39062076

[32] van Eijck CWF, Mustafa DAM, Vadgama D, de Miranda N, Groot Koerkamp B, van Tienhoven G, et al. Enhanced antitumour immunity following neoadjuvant chemoradiotherapy mediates a favourable prognosis in women with resected pancreatic cancer. Gut. 2024;73(2):311–24. 37709493 10.1136/gutjnl-2023-330480 PMC10850691

[33] Bankhead P, Loughrey MB, Fernandez JA, Dombrowski Y, McArt DG, Dunne PD, et al. QuPath: open source software for digital pathology image analysis. Sci Rep. 2017;7(1):16878. 29203879 10.1038/s41598-017-17204-5 PMC5715110

[34] Viallard JF, Lescure M, Oksenhendler E, Blanco P, Visentin J, Parrens M. STAT expression and TFH1 cells in CVID granulomatosis and sarcoidosis: immunological and histopathological comparisons. Virchows Arch. 2024;484(3):481–90. 37924346 10.1007/s00428-023-03684-6

[35] Cinetto F, Scarpa R, Carrabba M, Firinu D, Lougaris V, Buso H, et al. Granulomatous lymphocytic interstitial lung disease (GLILD) in common variable immunodeficiency (CVID): a Multicenter Retrospective Study of patients from Italian PID Referral centers. Front Immunol. 2021;12:627423. 33777011 10.3389/fimmu.2021.627423 PMC7987811

[36] Boursiquot JN, Gerard L, Malphettes M, Fieschi C, Galicier L, Boutboul D, et al. Granulomatous disease in CVID: retrospective analysis of clinical characteristics and treatment efficacy in a cohort of 59 patients. J Clin Immunol. 2013;33(1):84–95. 22986767 10.1007/s10875-012-9778-9

[37] Perlman DM, Sudheendra MT, Racilla E, Allen TL, Joshi A, Bhargava M. Granulomatous-lymphocytic interstitial lung Disease Mimicking Sarcoidosis. Sarcoidosis Vasc Diffuse Lung Dis. 2021;38(3):e2021025. 34744421 10.36141/svdld.v38i3.11114 PMC8552568

[38] Skytthe MK, Graversen JH, Moestrup SK. Targeting of CD163(+) macrophages in Inflammatory and Malignant diseases. Int J Mol Sci. 2020;21(15). 10.3390/ijms21155497 PMC7432735 32752088

[39] Granfeldt A, Hvas CL, Graversen JH, Christensen PA, Petersen MD, Anton G, et al. Targeting dexamethasone to macrophages in a porcine endotoxemic model. Crit Care Med. 2013;41(11):e309–18. 23928834 10.1097/CCM.0b013e31828a45ef

[40] Graversen JH, Svendsen P, Dagnaes-Hansen F, Dal J, Anton G, Etzerodt A, et al. Targeting the hemoglobin scavenger receptor CD163 in macrophages highly increases the anti-inflammatory potency of dexamethasone. Mol Ther. 2012;20(8):1550–8. 22643864 10.1038/mt.2012.103 PMC3412497

[41] Thomsen KL, Moller HJ, Graversen JH, Magnusson NE, Moestrup SK, Vilstrup H, Gronbaek H. Anti-CD163-dexamethasone conjugate inhibits the acute phase response to lipopolysaccharide in rats. World J Hepatol. 2016;8(17):726–30. 27330681 10.4254/wjh.v8.i17.726 PMC4911506

[42] Kristiansen M, Graversen JH, Jacobsen C, Sonne O, Hoffman HJ, Law SK, Moestrup SK. Identification of the haemoglobin scavenger receptor. Nature. 2001;409(6817):198–201. 11196644 10.1038/35051594

[43] Maglione PJ, Gyimesi G, Cols M, Radigan L, Ko HM, Weinberger T et al. BAFF-driven B cell hyperplasia underlies lung disease in common variable immunodeficiency. JCI Insight. 2019;4(5). 10.1172/jci.insight.122728 PMC6483510 30843876

[44] Lee NS, Barber L, Akula SM, Sigounas G, Kataria YP, Arce S. Disturbed homeostasis and multiple signaling defects in the peripheral blood B-cell compartment of patients with severe chronic sarcoidosis. Clin Vaccine Immunol. 2011;18(8):1306–16. 21653741 10.1128/CVI.05118-11 PMC3147362

