# Supplementary material for: Granulomas in Common Variable Immunodeficiency Display Different Histopathological Features Compared to Other Granulomatous Diseases
Source: J Clin Immunol. 2024 Oct 7;45(1):22. doi: 10.1007/s10875-024-01817-3 (PMC11458708; doi:10.1007/s10875-024-01817-3)
Supplement: Supplementary file 1 — Supplementary Material 1 [file 10875_2024_1817_MOESM1_ESM.docx]

**Granulomas in Common Variable Immunodeficiency display different histopathological features compared to other granulomatous diseases**

Astrid C. van Stigt^1,2^, Jan H. von der Thüsen^3^, Dana A.M. Mustafa^3,4^ ,Thierry P.P. van den Bosch^3^, Karishma A. Lila^3^, Disha Vadgama^3,4,5^, Martin van Hagen^1,2^, Virgil A.S.H. Dalm^1,2^, Willem. A. Dik^1^, Hanna IJspeert^1^

Affiliations

1. Erasmus Medical Center, Laboratory of Medical Immunology, Department of Immunology, Rotterdam, The Netherlands.

2. Erasmus Medical Center, Department of Internal Medicine, Division of Allergy and Clinical Immunology, Rotterdam, The Netherlands.

3. Erasmus Medical Center, Department of Pathology and Clinical Bioinformatics, Rotterdam, The Netherlands.

4. Erasmus Medical Center, the Tumor Immuno-Pathology Laboratory, Department of pathology, Rotterdam, The Netherlands

5. Erasmus Medical Center, Department of Pulmonary Medicine, Rotterdam, The Netherlands.

**Extended methods**

*Histology scoring*

On HE stains performed on tissue slides (thickness: 4 μm) of the included biopsies, a systematic and semi-quantitative histology scoring method was used to assess similarities and differences among biopsies. Parameters included: 1) organ location biopsy, 2) original tissue structure affected, 3) presence of granulomas, 4) presence of micro granulomas, 5) presence of large granulomas, 6) average diameter of five solitary granulomas, 7) presence of clustered granulomas, 8) presence of solitary granulomas, 9) presence of well-circumscribed granulomas, 10) presence of confluent granuloma areas, 11) presence of necrotic areas in granulomas, 12) presence of multinucleated giant cells, 11) number of multinucleated giant cells / granuloma, 12) presence of foreign body giant cell, 13) presence of touton giant cells, 14) presence of Langhans giant cells, 15) presence of asteroid body giant cells, 16) cell infiltration into granuloma structure (not surrounding), 18) presence of fibrosis in granuloma, 19) presence of fibrosis surrounding the granuloma (ring). Scoring was done numerically, with 0 = not present, 1=limited presence, 2 = frequent presence, 3= present throughout slide. Biopsies were scored in a blinded and randomized fashion by two independent observers, namely the first author (AcS) and the involved pathologist (JvT). Following unblinding, the findings on the biopsies and histological scores per disease group were compared. Each disease group's scores for histology hallmarks (6 slides per group) were analyzed, showing median, upper, and lower limits (Table 1). Statistical analysis was performed with 2way anova, with multiple comparisons test correction using Tukey’s test, comparing the mean of each disease group per histology hallmark. As to compare granuloma size per disease group, the diameter of 30 randomly selected granulomas for each disease group were pulled, with Kruskal-Wallis with Dunn’s multiple comparison correction to test for significance; significance level set 0.05. * = P ≤ 0.05, ** = P ≤ 0.01, *** = P ≤ 0.001, **** = P ≤ 0.0001. For the discriminative capacity of granuloma size, shown in Figure 1 and Table 2, receiver operator comparison (ROC) analysis was performed with indicated sensitivity, specificity and Likelihood ratio (LR) with the associated cutoff size of the granulomas.

*Spatial Protein analysis Assay*

For the spatial protein analysis assay, FFPE biopsies were sectioned in slices of 5μm, and transferred on super frost glass slides. Antigen retrieval and antibody incubation was done following the manufacture manual (NanoString Technology©, Seattle, WA, USA)(1).

The FFPE sections were stained with a mixture of selected commercially available Nanostring detection panels (see section DSP detection panels), covering 62 protein targets, and three morphological markers. Morphological markers included CD45 (Alexa flour 594 conjugated; #NBP2-34528AF594, Novus) as pan leukocyte marker, CD68 (Alexa Flour 532 conjugated;# NBP2-34587AF532, Novus) for monocytes/macrophages, and SYTO13 (# S7575, Thermofisher) for nucleus staining. Staining of protein target panels was performed simultaneously, together with staining for the morphological markers. Per included patient sample, three granulomas per slide were selected. We aimed to select granulomas as distant from each other as possible. Of each granuloma, the CD68-rich center and an adjacent surrounding area that did not contain any other granulomatous lesion were selected. This resulted in total of 144 ROIs (4 disease groups, 6 patients per group, 3 granulomas per patient with 2 areas per granuloma). Granulomas that were suspected of necrosis were not used for ROI selection. Also, we did not select granuloma areas containing multinucleated giant cells for technical reasons. Protein expression profiles were analyzed using GeoMx DSP and nCounter analysis (NanoString Technologies, Seattle, WA, USA). In total, 144 ROIs where selected and protein counts of 62 targets were harvested on subsequent days. Probe quantification on the nCounter was performed on one single run for all 144 ROIs. Protein count analysis was performed using the Nanostring GeoMx Platform (NanoString Technology©, Seattle, WA, USA)(1). and graph pad software version 8.0.1.

*DSP detection panels, normalization and background correction*

We used immunology detection panels offered by Nanostring, targeting 62 proteins in total (Table S1). The following detection panels were selected: Immune cell profiling core (Β-2-microglobulin, CD11c, CD20, CD3, CD4, CD45, CD56, CD68, CD8, CTLA4, pan-cytokeratin, fibronectin, GZMB, HLA-DR, Ki-67, PD-1. PD-L1, SMA), Immune activation status module (CD127, CD25, CD27, CD40, CD44, CD80, ICOS, PD-L2), Immune cell typing module (CD14, CD163, CD34, CD45RO, CD66b, FAP-α, FOXP3), PI3K-AKT module (Pan-AKT, MET, phospho-Akt1, phospho-GSK3B, phospho-tuburin, phosphor-GSK3A/ phosphor-GSK3B, INPP4B, PLCG1, phospho-PRAS40), MAPK module (EGFR, pan-RAS, BRAF, phospho-c-RAF, phospho-JNK, phospho-MEK1, phospho-p38 MAPK, phospho-p44/42 MAPK ERK1/ERK2, p44/42 MAPK ERK1/2, phospho-p90 RSK). For normalization H3 and S6 were selected due to their homogenous expression throughout all ROIs . Background correction was performed by subtracting the expression of Mouse (Ms)IgG2a from the expression of all targets per ROI

*Final protein count spatial protein analysis*

After normalization using Histone H3 and S6, and BGC using MsIgG2a, corrected protein counts were used for final analyses, including 72 granulomas and 72 surrounding ROIs. Per each of the 6 patients per disease group, 3 granulomas with 3 adjacent surroundings were used. For the volcano plots showing relative protein expression, the available analysis pipeline of GeoMx Nanostring was used, with statistical testing using linear mixed model followed by BH correction. In the volcano plots, significantly different protein targets with uncorrected P values are shown.

For the absolute protein counts of selected protein targets, the protein expressions were considered non-parametric. Statistical testing was performed using Mann-Whitney test for inter-disease differences between granuloma versus surrounding per disease, and Kruskall-Walis with Dunns post hoc test to correct for multiple testing for comparing granuloma or surrounding protein expression between the four diseases.

*IF multiplex modules and granuloma annotation*

On three tissue slides per biopsy (thickness 4 μm), subsequently cut after slide preparation for the first HE staining and DSP assay, three Immunofluorescence multiplex assays were performed. Additionally, the final tissue slide was used for a second HE stain. The following IF multiplex were run: Multiplex 1: CD68, CD163, MPO, phospho-ERK1/2, FAP-alpha, Multiplex 2: SMA, FAP-alpha, PDL1, CD11c, CD68, Multiplex 3: CD3, CD4, foxp3, CD20, PD-1. Per slide, 5 granulomas and their adjacent surroundings were annotated. To this end, 10 annotated areas per slide were used for further analysis. For accurate granuloma annotation, HE stains were used as a reference slide. Additionally, CD68 aided in granuloma selection, which was only possible for multiplex 1 and 2. For multiplex 3, CD3 aided in granuloma selection.

*Automated Multiplex Immunofluorescent Staining*

Multiplex immunofluorescence stains were done by automated multiplex IF using the Ventana Benchmark Discovery (Ventana Medical Systems Inc.). In brief, following deparaffinization and heat-induced antigen retrieval with CC1 (#950-500, Ventana) for 40 minutes at 97°C, the tissue samples were incubated firstly with MPO for 20 minutes at 37˚C followed by detection with omnimap anti-rabbit HRP (#760-4311, Ventana) for 20 minutes followed by visualization with R6G for 8 minutes (#760-244, Ventana). Antibody denature step was performed using CC2 (#950-123, Ventana) for 20 minutes at 100˚C. Secondly, CD68 was incubated for 32 minutes at 37˚C followed by detection with omnimap anti-mouse HRP (#760-4310, Ventana) followed by visualization with DCC for 4 minutes (#760-240, Ventana). An antibody denaturation step was performed using CC2 (#950-123, Ventana) for 8 minutes at 100˚C. Thirdly, CD163 was incubated for 32 minutes at 37 ˚C followed by detection with omnimap anti-mouse HRP (#760-4310, Ventana) for 20 minutes followed by visualization with Red610 (#760-245) for 8 minutes. Fourthly, p-ERK was incubated for 32 minutes at 37˚C followed by detection with omnimap anti-rabbit HRP (#760-4311, Ventana) followed by visualization with Cy5 (#760-243) for 8 minutes. Lastly, FAP was incubated for 60 minutes at 37˚C followed by detection with omnimap anti-rabbit HRP (#760-4311, Ventana) for 20 minutes followed by visualization with FAM (#760-243, Ventana) for 8 minutes.

In brief for Panel 2, following deparaffinization and heat-induced antigen retrieval with CC1 (#950-500, Ventana) for 40 minutes at 97°C, the tissue samples were incubated firstly with CD11c for 48 minutes at 37˚C followed by detection with omnimap-rabbit HRP (#760-4311, Ventana) for 20 minutes followed by visualization with R6G for 8 minutes (#760-244, Ventana). Antibody denature step was performed using CC2 (#950-123, Ventana) for 20 minutes at 100˚C. Secondly, CD68 was incubated for 20 minutes at 37˚C followed by detection with omnimap anti-mouse HRP (#760-4310, Ventana) followed by visualization with DCC for 4 minutes (#760-240, Ventana). An antibody denaturation step was performed using CC2 (#950-123, Ventana) for 8 minutes at 100˚C. Thirdly, FAP was incubated for 60 minutes at 37 ˚C followed by detection with omnimap anti-rabbit HRP (#760-4311, Ventana) for 20 minutes followed by visualization with Red610 (#760-245) for 8 minutes. Fourthly, PD-L1 was incubated for 60 minutes at 37˚C followed by detection with omnimap anti-rabbit HRP (#760-4311, Ventana) followed by visualization with Cy5 (#760-243) for 4 minutes. Lastly, SMA was incubated for 60 minutes at 37˚C followed by detection with omnimap anti-mouse HRP (#760-4310, Ventana) for 8 minutes followed by visualization with FAM (#760-243, Ventana) for 4 minutes.

In brief for Panel 3, following deparaffinization and heat-induced antigen retrieval with CC1 (#950-500, Ventana) for 40 minutes at 97°C the tissue samples were incubated firstly with CD4 for 24 minutes at 37˚C followed by detection with omnimap-rabbit HRP (#760-4311, Ventana) for 16 minutes followed by visualization with R6G for 8 minutes (#760-244, Ventana). Antibody denature step was performed using CC2 (#950-123, Ventana) for 20 minutes at 100˚C. Secondly, FOXP3 was incubated for 60 minutes at 37˚C followed by detection with omnimap anti-mouse HRP (#760-4310, Ventana) followed by visualization with DCC for 4 minutes (#760-240, Ventana). Antibody denature step was performed using CC2 (#950-123, Ventana) for 8 minutes at 100˚C. Thirdly, CD3 was incubated for 24 minutes at 37 ˚C followed by detection with omnimap anti-rabbit HRP (#760-4311, Ventana) for 20 minutes followed by visualization with Red610 (#760-245) for 8 minutes. Fourthly, PD-1 was incubated for 32 minutes at 37˚C followed by detection with omnimap anti-mouse HRP (#760-4310, Ventana) followed by visualization with Cy5 (#760-243) for 8 minutes. Lastly, CD20 was incubated for 40 minutes at 37˚C followed by detection with omnimap anti-mouse HRP (#760-4310, Ventana) for 20 minutes followed by visualization with FAM (#760-243, Ventana) for 4 minutes.

Slides were incubated in PBS with DAPI for 15 minutes and covered with anti-fading medium (DAKO, S3023). Antibody information and clonality can be found in Table S2.

*Digital Quantification IF stains using Qupath*

After staining, the slides were scanned with a ZEISS Axio lmager 2.0 fluorescence microscope using a 20x magnification. The scanned slides were analyzed using QuPath: Quantitative Pathology and Bioimage analysis software, version 0.4.3(2). The specific region of interest (ROI) was marked by manual annotations. The 'Cell detection' command identified nuclei via the DAPI signal, utilizing consistent settings for all slides and batches. Qupath’s simple threshold method was used to classify each fluorescently labeled cellular marker and calculate the number of positive cells based on thresholding per tissue slide. A classifier was created for each immunofluorescence multiplex setup, combining DAPI and the five included marker channels that were run simultaneously prior to protein count data extraction. These measurements were used for further statistical analysis. Positive cell counts were normalized on total cells detected per annotated area. Quintuple positive cells were excluded from analysis. Analyses were run using Excel and GraphPad Prism version 8.0.1. Due to the small number of samples, the dataset was considered as nonparametric with 2way anova and Tukey's multiple comparisons test used for statistical analysis and multiple testing.

**Supplemental tables**

**Supplemental Table S1. DSP targets per Nanostring module**

| **Controls** | **Immune Activation Status Panel** | **Immune Cell Profiling Panel** | **Immune Cell Typing Panel** | **MAPK Signaling Panel** | **PI3K/AKT Signaling Panel** |
| --- | --- | --- | --- | --- | --- |
| Rb IgG | CD127 | PD-1 | CD45RO | BRAF | Phospho-AKT1 (S473) |
| Ms IgG1 | CD25 | CD68 | FOXP3 | EGFR | Phospho-GSK3B (S9) |
| Ms IgG2a | CD80 | HLA-DR | CD34 | Phospho-c-RAF (S338) | Phospho-GSK3A (S21)/Phospho-GSK3B (S9) |
| Histone H3 | ICOS | Ki-67 | CD66b | Phospho-JNK (T183/Y185) | INPP4B |
| S6 | PD-L2 | Beta-2-microglobulin | FAP-alpha | Phospho-MEK1 (S217/S221) | PLCG1 |
| GAPDH | CD40 | CD11c | CD14 | Phospho-p38 MAPK (T180/Y182) | Phospho-PRAS40 (T246) |
|  | CD44 | CD20 | CD163 | Phospho-p44/42 MAPK ERK1/2 (T202/Y204) | Phospho-Tuberin (T1462) |
|  | CD27 | CD3 |  | pan-RAS | Pan-AKT |
|  |  | CD4 |  | p44/42 MAPK ERK1/2 | MET |
|  |  | CD45 |  | Phospho-p90 RSK (T359/S363) | Phospho-AKT (T308) |
|  |  | CD56 |  |  |  |
|  |  | CD8 |  |  |  |
|  |  | CTLA4 |  |  |  |
|  |  | GZMB |  |  |  |
|  |  | PD-L1 |  |  |  |
|  |  | PanCk |  |  |  |
|  |  | SMA |  |  |  |
|  |  | Fibronectin |  |  |  |

**Supplemental Table S2. Antibody information**

| **Antibody** | **Type** | **Concentration/Dilution** | **Company** | **Clone** |
| --- | --- | --- | --- | --- |
| MPO | Anti-Rabbit | 7.00 µl/ml | Ventana | Polyclonal |
| CD68 | Anti-Mouse | 0.4 µl/ml | Ventana | KP-1 |
| CD163 | Anti-Mouse | 0.28 µl/ml | Ventana | MRQ-26 |
| p-ERK | Anti-Rabbit | 1/1000 | Sigma | Monoclonal |
| FAP | Anti-Rabbit | 1/100 | Abcam | Monoclonal |
| CD11c | Anti-Rabbit | RTU | BioSB | EP157 |
| PD-L1 | Anti-Rabbit | 7 µl/ml | Ventana | SP142 |
| SMA | Anti-Mouse | 1:2500 | BioSite | BS66 |
| CD4 | Anti-Rabbit | 2.5 µl/ml | Ventana | SP35 |
| FOXP3 | Anti-Mouse | 1/100 | Invitrogen | 236a-e7 |
| CD3 | Anti-Rabbit | 0.4 µl/ml | Ventana | 2GV6 |
| PD-1 | Anti-Mouse | 4. µl/ml | Ventana | NAT105 |
| CD20 | Anti-Mouse | 0.3 µl/ml | Ventana | L26 |

**Supplemental Figure legends**

*Supplemental Figure S1*

Clustering overview of spatial protein analysis performed. Heat map analysis of all 62 proteins is shown, with different subgroup cluster analyses to observe clustering based on the indicated group label.

*Supplemental Figure S2*

Absolute counts of expression of A) MAPK pathway targets granuloma and surrounding all four diseases. Statistical testing was performed by Kruskall-Walis with Dunn’s multiple comparison correction, * = P ≤ 0.05, ** = P ≤ 0.01, *** = P ≤ 0.001.

*Supplemental Figure S3*

Different FAPα stains on IF. A) Immunofluorescence single stains showing FAPα ring formation surrounding the granulomas, of the two IF multiplex assays that included FAPα.

**Extended methods references**

1. van Eijck CWF, Mustafa DAM, Vadgama D, de Miranda N, Groot Koerkamp B, van Tienhoven G, et al. Enhanced antitumour immunity following neoadjuvant chemoradiotherapy mediates a favourable prognosis in women with resected pancreatic cancer. Gut. 2024;73(2):311-24.

2. Bankhead P, Loughrey MB, Fernandez JA, Dombrowski Y, McArt DG, Dunne PD, et al. QuPath: Open source software for digital pathology image analysis. Sci Rep. 2017;7(1):16878.
