# Supplementary figures and images for: Granulomas in Common Variable Immunodeficiency Display Different Histopathological Features Compared to Other Granulomatous Diseases
Source: J Clin Immunol. 2024 Oct 7;45(1):22. doi: 10.1007/s10875-024-01817-3 (PMC11458708; doi:10.1007/s10875-024-01817-3)

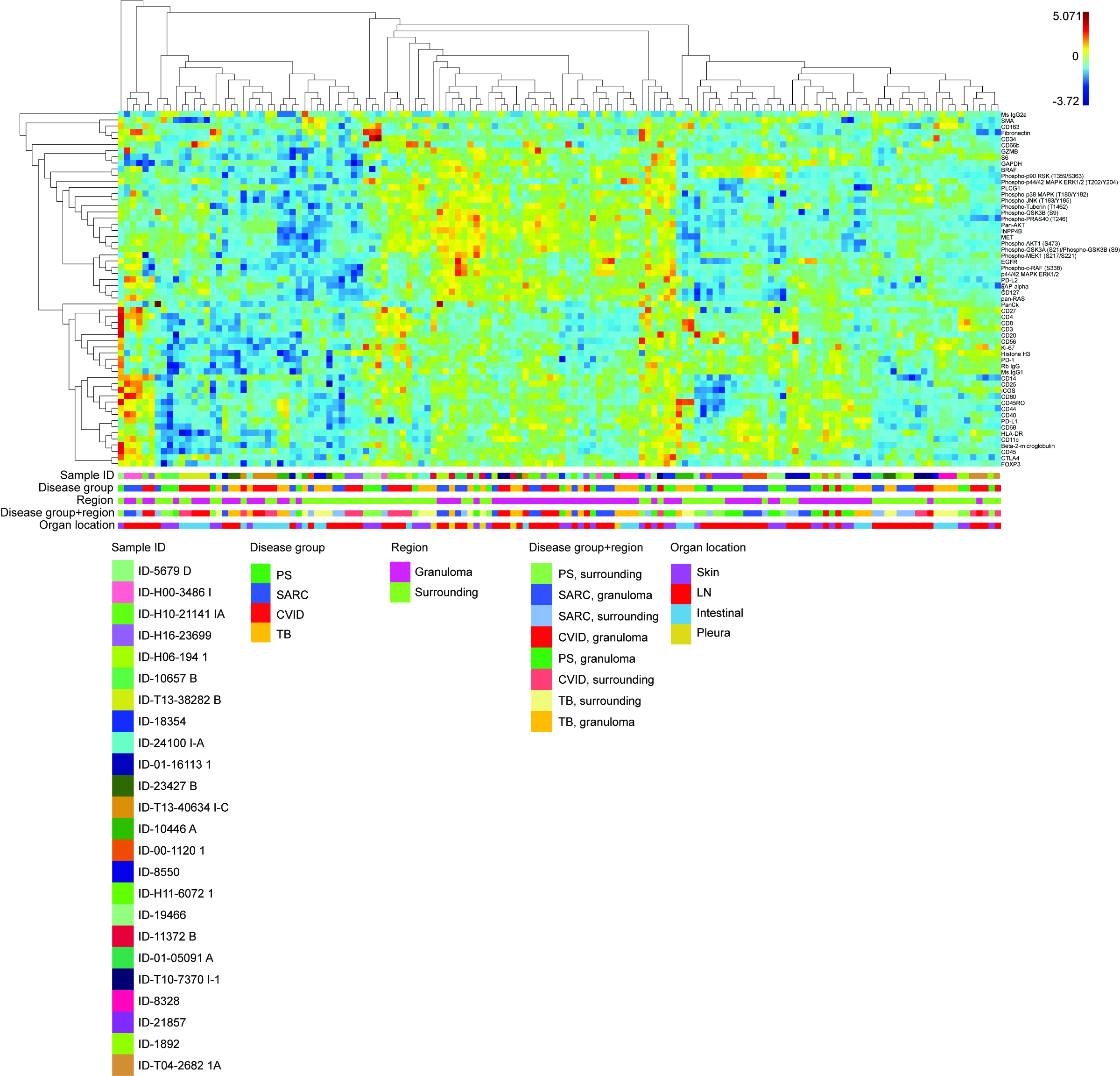

Supplement: Supplementary file 2 — Supplementary Material 2 [file 10875_2024_1817_MOESM2_ESM.tif]

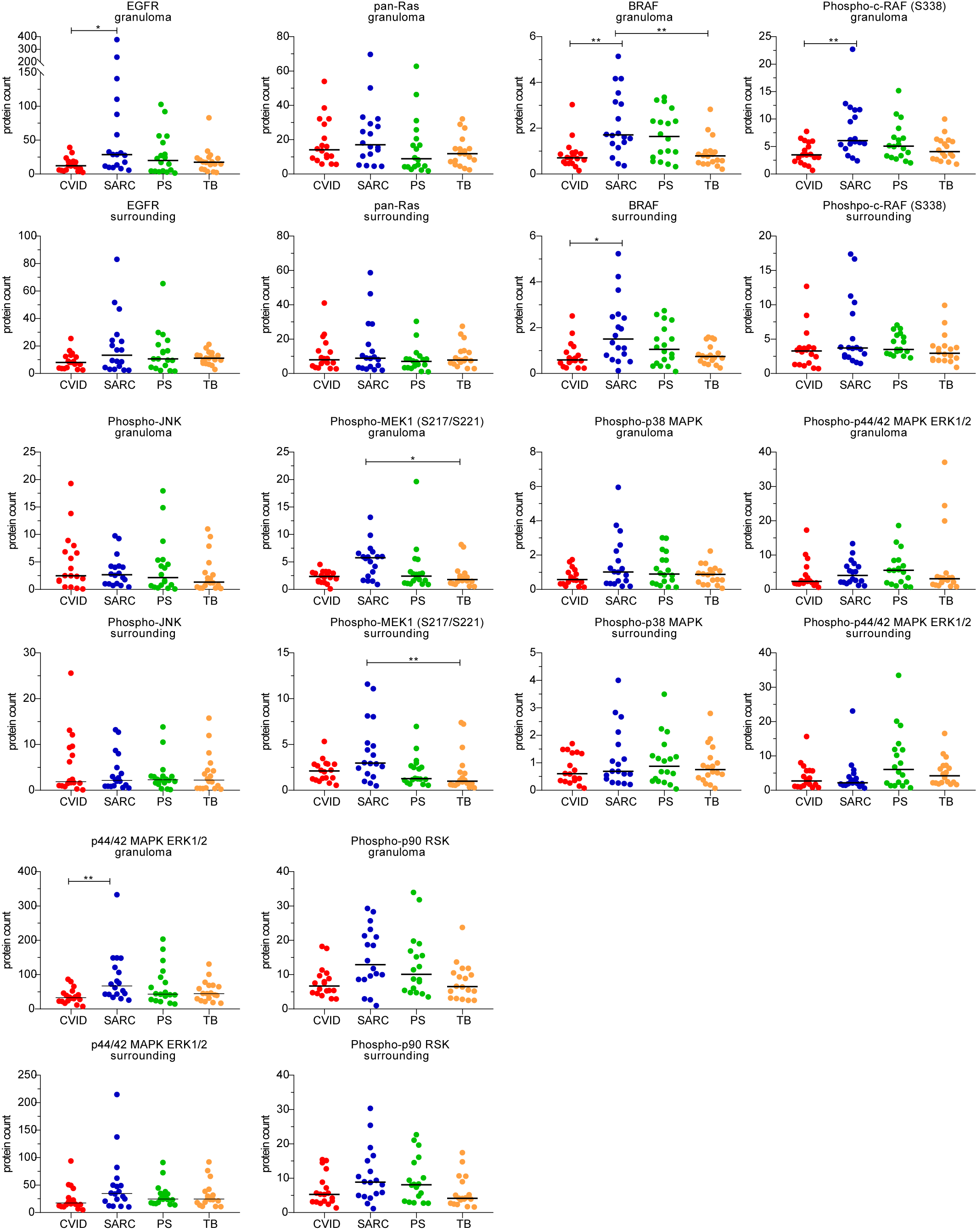

Supplement: Supplementary file 3 — Supplementary Material 3 [file 10875_2024_1817_MOESM3_ESM.tif]

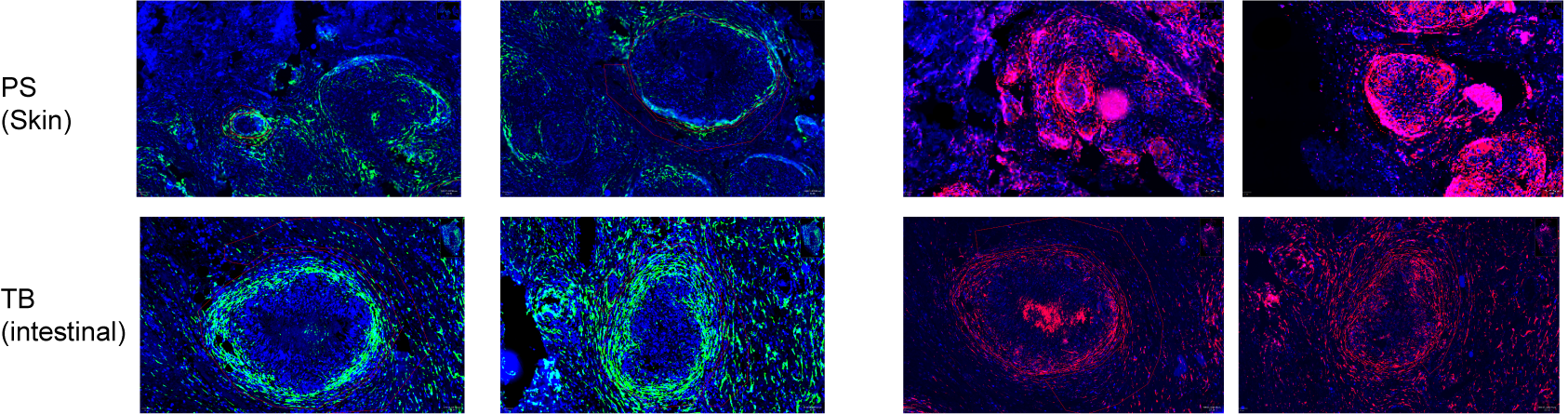

Supplement: Supplementary file 4 — Supplementary Material 4 [file 10875_2024_1817_MOESM4_ESM.tif]
